# Effect of Single-Dose Methotrexate Treatment on Ovarian Reserve in Women with Ectopic Pregnancy Undergoing Infertility Treatment: A Single-Center Experience

**DOI:** 10.22074/ijfs.2020.5938

**Published:** 2020-02-25

**Authors:** Mahboobeh Shirazi, Parichehr Pooransari, Neda Hajiha, Zeinab Shaker, Mohadeseh Ghazi, Fatemeh Davari Tanha, Batool Ghorbani Yekta, Marjan Ghaemi

**Affiliations:** 1 Maternal, Fetal and Neonatal Research Center, Tehran, Iran; 2 Breast Feeding Research Center, Tehran University of Medical Sciences, Tehran, Iran; 3 Shohada Hospital, Shahid Beheshti University of Medical Sciences, Tehran, Iran; 4 Young Researchers and Ethics Club, Tehran Medical Science Branch, Islamic Azad University, Tehran, Iran; 5 Herbal Pharmacology Research Center, Tehran Medical Science Branch, Islamic Azad University, Tehran, Iran; 6 Kamali Hospital, Alborz University of Medical Sciences, Karaj, Iran

**Keywords:** Anti-Mullerian Hormone, Assisted Reproductive Techniques, Ectopic Pregnancy, Methotrexate, Ovarian Reserve

## Abstract

**Background:**

The aim of this study was evaluation of the impact of single-dose methotrexate (MTX) treatment on ovarian reserve in women with ectopic pregnancy (EP) undergoing infertility treatment in Iranian population.

**Materials and Methods:**

This prospective cohort study was done between March 2015 and March 2017 in Tehran General Women Hospital, Tehran, Iran. We enrolled 20 patients with EP who conceived during infertility treatment and received a single-dose MTX (50 mg/m^2^) intramuscularly. Serum anti-Mullerian hormone (AMH), 17 beta-estra diol (E2), luteinizing hormone (LH), follicle-stimulating hormone (FSH) and antral follicle count (AFC) on transvaginal ultrasonography, were evaluated before and 8 weeks after administration of MTX.

**Results:**

AMH did not significantly vary after the administration of MTX, compared to before treatment value (P=0.36). FSH, E2 and AFC changes were not statistically significant, while increment of LH was significant (P=0.02).

**Conclusion:**

Results indicated that single-dose MTX treatment did not reduce ovarian reserve in women with EP. Further randomized controlled clinical trial studies with larger sample sizes, by using multiple dosages of MTX, and with long-term follow up are suggested to be done.

## Introduction

Ectopic pregnancy (EP) occurs when a fertilized egg implants somewhere other than the main cavity of the uterus. EP cannot continue as a normal pregnancy. EP comprises about 1.6% of all pregnancies and it is a potential leading cause of pregnancy-related mortality in the first trimester of pregnancy (1).

Laparoscopy is the gold standard for managing EP (2). Conservative management can be medical or expectant, however, watchful selection and counseling are important, as non-surgical approach may expose the women to the risk of tubal rupture. The most commonly used drug for medical management is methotrexate (MTX). It is an antimetabolite drug that acts on actively growing cells (3). This mechanism effectively treats EP but it is supposed that MTX has an impact on fertility by targeting the actively dividing granulosa cells (GC) within the ovary. This, in turn, may decrease ovarian reserve and further responsiveness (4).

Anti-Mullerian hormone (AMH) is an endocrine marker considered for assessing the ovarian reserve and it is not affected by gonadotropins (5). Also, luteinizing hormone (LH) and follicle stimulation hormone (FSH) were assessed during the course of the treatment and monitoring. AMH values can be measured at any point during the menstrual cycle without the need for a sonogram, which makes it a unique and specific test in evaluation of ovarian reserve (6). Assisted reproductive techniques (ART) may be an opportunity to study the effect of MTX on ovarian reserve. The novelty of our study was related to the time interval between measurements and assessment of the hormonal levels after administration of only one dose of MTX. The present study was conducted to assess the effect of single-dose MTX on ovarian reserve by measuring AMH in women with EP undergoing infertility treatment in Iranian population.

## Materials and Methods

This prospective cohort study was conducted from 2015 to 2017 in Tehran General Women Hospital, an educational hospital affiliated with Tehran University of Medical Sciences (TUMS), Tehran, Iran. The study was approved by the Ethical Board Committee of Tehran University of Medical Sciences by number 90-0339-14127. Patients were thoroughly informed of the experiment and fully consented to taking part in the study

### Sample selection

Patients who referred to our hospital with suspicious EP, were assessed for eligibility. AFC, AMH, (FSH and LH on the 3rd day of the cycle) and 17 beta estradiol (E2) in mid cycle were assessed before recruiting in the ART cycle. For baseline assessment, beta-human chorionic gonadotropin (β-hCG) concentration was measured and trans-vaginal ultrasound was performed to evaluate the pregnancy sac in spontaneous course and at baseline before any intervention. The diagnosis of EP was made based on an increasing serum β-hCG concentration (>2000 mlU/ml) and no intrauterine sac visualized by trans-vaginal ultrasound after 6 weeks of gestational age. The unnecessary tests are not mentioned. All tests were done in a single lab in the reference hospital.

Eligible subjects were adult women with at least 18 years of age with history of infertility that had previously received *in vitro* fertilization (IVF) and received single dose MTX (50 mg/m^2^) intramuscularly for mangling EP.

 Eligibility for MTX administration included: stable homodynamic status, the size of ectopic mass below 4 cm on ultrasound examination, unruptured EP and no contraindication either relative or absolute for MTX use. Serum concentration of β-hCG >5000 mlU/ml and fetal heart activity were relative contraindications for nonsurgical management of EP. Absolute contraindications were chronic liver disease, pre-existing blood dyscrasias, pulmonary disease, peptic ulcer disease and immunodeficiency. In addition, the participants who had sensitivity to MTX, or were breastfeeding, were not included for MTX therapy (7). Patients were thoroughly informed about the experiment and fully consented to taking part in the study.

### Intervention and outcome

Plasma levels of 17 beta-estradiol (in mid cycle), LH and FSH (on the 3rd day of the cycle) as well as AMH were measured at baseline (definitely before MTX administration) and 8 weeks after treatment with MTX. Selection of the time point (i.e. after eight weeks) was according to our pilot study that showed the highest alteration at this time. Antral follicle count (AFC) was estimated by trans-vaginal ultrasound before and after the study. These markers had been checked in the same laboratory before pregnancy.

### Statistical analysis

Statistical analysis was done by SPSS version 19.0 software (IBM SPSS Statistics, USA). Mean ± SD and numbers (%) were calculated for continuous and categorical variables, respectively. T test for paired observation was used (after running KolmogorovSmirnov test reassurance for parametric distribution) to evaluate any differences in AMH, LH, FSH, AFC and E2 values between the pre-pregnancy values and those obtained 8 weeks after MTX administration. A P≤0.05 was considered statistically significant.

Sample size was calculated by Cochran’s formula. Given the probability of 1.6% of EP (in normal population) of which 35% are eligible for receiving medical treatment, the estimated sample size was 20. The sample size was determined by a pilot study done on ten subjects before initiation of the main study.

## Results

Twenty patients were recruited and all of them were followed until the end of the study. None of our cases needed extra dose of MTX nor needed an emergent surgery due to ruptured EP. No serious adverse effect was reported during the study. None of our cases had persistent EP. In other word, all patients were cured both clinically and according to laboratory tests.

The mean (± SD) age of patients was 30.9 ± 5.37 years (range: 21 to 43 years old). Table 1 illustrates the age and obstetrics background of the participants. Two patients had a history of EP. None of the participants had heterotopic pregnancy.

**Table 1:** Age and obstetrics background of the participants


Variables		n=20

Age (Y)		30.9 ± 5.37
Gravidity		
	1	4 (20)
	2	7 (35)
	3	6 (30)
	4	3 (15)
Parity		
	0	10 (50)
	1	6 (30)
	2	4 (20)
Abortion status		
	0	10 (50)
	1	8 (40)
	2	2 (10)
BMI (Kg/m^2^)		27.1 ± 4.7


Data are presented as mean ± SD or n (%). BMI; Body mass index.

The mean (± SD) of AMH levels at baseline and after 8 weeks were 9.5 ng/ml (± 4.23) and 9.15 ng/ml (± 4.24), respectively which were not statistically significantly different (P=0.36). For FSH, E2 and AFC, there was a nonstatistically significant difference between baseline value and the value obtained 8 weeks after MTX administration. However, the mean (± SD) LH values were 6.63 IU/l (± 3.03) and 8.1 IU/l (± 2.63) at baseline and 8 weeks after MTX administration, respectively (P=0.02). Table 2 compares lab data and AFC, before and after EP treating by MTX.

**Table 2:** Comparison lab data and AFC, before and after EP treated by MTX


Variables	Pre-MTX	Post-MTX	P value

FSH (mIU/ml)	6.6 ± 3.1	8.2 ± 4.9	0.1
LH (IU/L)	6.6 ± 3.0	8.1 ± 2.6	0.02
AMH (ng/ml)	9.5 ± 4.2	9.1 ± 4.2	0.36
AFC	9.1 ± 2.0	8.6 ± 2	0.49
E2 (pg/ml)	21.9 ± 20.1	16.3 ± 13.5	0.15


Data are presented as mean ± SD. AFC; Antral follicle count, EP; Ectopic pregnancy, MTX; Methotrexate, FSH; Folliclestimulating hormone, LH; Luteinizing hormone, AMH; Anti-Mullerian Hormone, and E2; 17-beta estradiol.

## Discussion

Our study demonstrated that medical management of EP using MTX does not have adverse effects on ovarian reserve in infertile women undergoing various types of ART. EP management by using MTX is safe and effective in carefully selected patients. It is beneficial in cases with no tendency for surgery (7) and helps to decline the rate of surgery. Indeed, it is safe for a later pregnancy. The fetal exposure to MTX from maternal organs is considered to be low and the outcomes of pregnancies shortly after MTX therapy, are almost favorable (8).

Another important issue in management of patients is the maintenance of women’s fertility. Infertile women under infertility treatment, may already have compromised ovarian reserve (4). Furthermore, the data known about the effects of MTX on ovarian reserve and effectiveness of subsequent ART, is minimal.

Evaluation of ovarian reserve is important in assessment and treatment of infertility. Ovarian reserve will decline by age. The most commonly used approach to assess ovarian reserve, is the measurement of FSH and LH. However, AMH and inhibin-B are other biomarkers of ovarian reserve that are most popular since they provide direct determination of ovarian status (9). In this study, LH had significant difference despite narrow difference of values.

Ovarian reserve, ovarian responsiveness, or subsequent IVF outcomes was discussed in other studies and most of such reports did not reveal a significant difference in IVF cycle parameters or outcomes. Orvieto et al. (10) demonstrated no difference in FSH, ovarian stimulation characteristics, or oocytes retrieved in IVF patients before and after receiving MTX treatment for EP. Also, in another study on women undergone IVF/intra-cytoplasmic sperm injection (ICSI), no difference in AMH, stimulation parameters, oocytes retrieved, or number of embryos was found between before and after MTX administration (11).

Pregnancy rate after MTX administration was 36.4%, that is similar to normal rate showing no modification of the characteristics of the endometrial or follicles during IVF after MTX treatment for EP (12). In another study on patients who underwent an IVF cycle that resulted in an EP and patients treated with MTX, no adverse effect on ovarian reserve or ovarian responsiveness was found (4). Similarly, in our study, AMH, AFC and FSH as a main biomarker for ovarian function, did not change after MTX administration. Ohannessian et al. (13) in a meta-analysis, similarly demonstrated that comparisons between before and after treatment with MTX showed no statistically significant differences in the basal plasma FSH level, total gonadotrophin dose used for stimulation, duration of stimulation, and E2 level on the day ovulation was triggered.

While the evidence from human studies is limited, results of studies that assessed the effect of MTX on AMH as an ovarian reserve marker, are controversial. Dosages of MTX, sample size and follow up period length have been suggested as factors altering the results.

Our study had some limitations that should be mentioned. It was a retrospective study with small sample size. Considering our results which were similar to those of other studies, it was not possible to definitely prove that MTX has no adverse effect on ovarian reserve and responsiveness in Iranian population.

The strength of our study was evaluation of AMH during evaluation of ovarian reserve that was absent in a similar study by Boots et al. (4). Nowadays, AMH is routinely measured in multiple occasions especially for infertility evaluation. Measurement of AMH is considered a highly effective approach of assessing ovarian reserve because of its independence of the menstrual cycle as well as the higher inter-cycle and intra-cycle reproducibility (8).

## Conclusion

Our results showed that single-dose MTX treatment in EP did not decrease the ovarian reserve in infertile women.
